# Intensive Swallowing Rehabilitation for Head and Neck Cancer Survivors With Chronic Dysphagia: Outcomes From a Prospective Multicentre Cohort Study With 6‐Month Follow‐Up

**DOI:** 10.1002/hed.70271

**Published:** 2026-04-10

**Authors:** J. I. Oldenbeuving, L. van der Molen, J. T. Groothuis, J. G. Kalf

**Affiliations:** ^1^ Radboud University Medical Center, Donders Institute for Brain, Cognition and Behaviour, Department of Rehabilitation Nijmegen the Netherlands; ^2^ Antoni Van Leeuwenhoek ‐ Netherlands Cancer Institute, Department of Head and Neck Oncology and Surgery Amsterdam the Netherlands

**Keywords:** head and neck cancer, intensive swallowing rehabilitation, long‐term dysphagia, speech language therapist, swallowing related quality of life

## Abstract

**Background:**

Chronic dysphagia persists in some head and neck cancer (HNC) survivors despite preventive exercises, negatively impacting quality of life. Evidence for effective rehabilitation strategies remains limited.

**Methods:**

This prospective cohort study included 34 HNC survivors (≥ 1 year post‐treatment) who completed a 3‐week intensive swallowing rehabilitation (ISR) program (15 sessions, 60 min/day). Outcomes were measured at baseline (T0), post‐treatment (T1), and 6‐month follow‐up (T2) using patient‐rated (VAS, MDADI, PSS‐HN), clinician‐rated (food level, AUS‐TOM), and objective swallowing outcomes (MSS, MSV, TOMASS).

**Results:**

ISR was feasible and safe (92% completion, no adverse events). Patient‐rated and most clinician‐rated outcomes improved significantly post‐ISR and remained stable at 6 months (VAS: *p* < 0.001; PSS‐HN: *p* < 0.001; food level: *p* = 0.002). MDADI and AUS‐TOM for solids further improved at follow‐up. Objective swallowing tests showed no clinically relevant change.

**Conclusion:**

ISR effectively improves normalcy of diet and quality of life in HNC survivors with chronic dysphagia, with benefits sustained at 6 months.

## 1 Introduction

Dysphagia is a well‐known complication of head and neck cancer (HNC), where swallowing impairments are mostly caused by adverse effects of medical treatment [1, 2]. The type and severity of dysphagia following medical treatment in HNC will depend upon the tumor stage, the location of the primary tumor, involved structures, type and extent of the medical treatment, and individual patient factors such as age, general health, and nutritional status [3, 4, 5, 6, 7]. The nutritional and social limitations caused by chronic swallowing difficulties can have a major negative impact on quality of daily life in HNC patients [8, 9, 10].

HNC patients treated with (chemo)radiotherapy [4, 10, 11] or (free flap) surgery [12, 13], may continue to experience swallowing difficulties. Years after medical treatment, HNC patients report swallowing problems focused on efficiency (normalcy of diet) rather than safety [4, 10, 11, 12, 13] One study, for instance, found that approximately 79% of HNC patients reported problems with solid foods around 20 months after (chemo)radiation, while only 20% experienced issues with liquids [4]. Another study, focusing on HNC patients 1 year after oral cancer surgery, showed that although the vast majority (94%) no longer needed a feeding tube, and only 10% showing signs of penetration or aspiration, those patients were still depended on a modified diet [12]. Even up to 6 years post‐(chemo)radiotherapy, research indicates that half of the HNC patients were unable to return to a normal diet [10]. These findings collectively suggest that, from a patient's perspective, the swallowing difficulties are more defined by inefficient than by unsafe swallowing.

Several studies have explored the use of preventive therapies in HNC patients to reduce long‐term effects on swallowing [14], showing promising results for specific treatment regimens. However, the overall effectiveness of an exercise protocol for the treatment of chronic dysphagia in HNC patients remains limited and there is insufficient evidence regarding timing, intensity or specific types of swallowing exercises [14].

For HNC patients who experience chronic dysphagia in HNC, despite preventive swallowing therapy in some cases, various swallowing treatments are available [14, 15, 16, 17, 18, 19, 20, 21]. These include regular swallowing exercises [21], respiratory exercises [15], respiratory‐swallow training [20], exercise regime with swallow exercise aid (SEA) [17], surgical interventions such as lipofilling [16] and very intensive swallowing treatments [18, 19]. Among these, the very intensive swallowing treatments have shown improvement in both swallowing safety and normalcy of diet. In contrast, the other interventions, if effective, showed primarily improvement on swallowing safety. The intensive treatments are based on the principles of the 3‐week McNeill dysphagia therapy program (MDTP) [22, 23, 24], which is not primarily designed for HNC patients. A 10‐week intensive swallowing program in 10 HNC patients [19] showed improvements on swallowing safety (based on videofluoroscopy), this program and a 3‐week bootcamp in 34 HNC patients both found an improvement in dysphagia‐related quality of life and normalcy of diet [18, 19]. However, neither study included a follow‐up period, leaving it unclear whether improvements sustain a longer period.

Therefore, the goal of our study was (1) to investigate whether a 3‐week intensive swallowing rehabilitation (ISR), tailored for HNC patients, is feasible and effective, (2) to understand the impact on dysphagia‐related quality of life, including normalcy of diet, and (3) whether improvements, if any, sustain during a 6‐month follow‐up period.

## 2 Methods

### 2.1 Study Design and Setting

This prospective multicenter cohort study of ISR was conducted at the outpatient clinic of the Department of Rehabilitation of the Radboud university medical center (Radboudumc, Nijmegen) and the Department of Head and Neck Oncology and Surgery of the Netherlands Cancer Institute (NKI, Amsterdam) between July 2020 and July 2022.

The study protocol was reviewed by the medical ethics committee (METC) of the Radboudumc and was considered as a non‐WMO study (registration #2020‐6710 (Radboudumc)). Furthermore, the Institutional Review Board (IRB) of the NKI reviewed and approved the study as non‐WMO (NKI registration IRBd21‐093). Informed consent was obtained from all participants.

### 2.2 Recruitment

In both centers, the HNC board members (e.g., head and neck surgeons, specialized nurses, etc.) were informed and requested to refer HNC patients to the speech and language therapist (SLT) within their centers. Additional recruitment occurred via the national HNC SLT working group and the Dutch patient association for head and neck cancer (Patiëntenvereniging Hoofd‐Hals (PVHH)). A study advertisement was published in the PVHH association's magazine. External participants could be referred and included after formal medical approval.

### 2.3 Participants

Participants were included if they met the following criteria: (1) head–neck cancer (irrespective of tumor type or medical treatment) patients with persistent dysphagia at least 1 year after medical treatment, (2) physically and cognitively capable and willing to complete ISR. Participants were excluded when scheduled medical interventions interfered with participation.

### 2.4 Data Collection

After written consent and intake (including videofluoroscopy), participants followed a 3‐week ISR program (15 sessions, 60 min/day). Data was collected at baseline (T0), immediately post ISR (T1) and 6 months post ISR (T2).

A local clinical assessment tool developed by Radboudumc was used, which included patient‐rated and clinician‐rated outcomes, and swallowing performance tests. This tool does not contain all patient‐rated outcomes; therefore, additional measures were collected separately, including the visual analogue scale (VAS), the M.D. Anderson Dysphagia Inventory (MDADI), and goal achievement. Together, these form the complete set of patient‐ and clinician‐rated outcomes described below.

Patient‐rated swallowing outcomes:

- Participants were asked to indicate their response to the question “How would you rate your swallowing?” by placing a mark on a VAS ranging from 0 (I can't swallow) to 100 (I have no swallowing problems).
- Dysphagia‐related quality of life was conducted by using the Dutch version of the MDADI [25]. The MDADI consists of 20 items and is divided into three subscales: emotional (participants affective response to swallowing disorder), functional (impact on daily activities) and physical (self‐perception of swallowing difficulties). MDADI scores range from 20 to 100, with highest scores representing a high dysphagia‐related quality of life.
- Social participation was measured by a subscales of the Performance Status Scale for Head and Neck Cancer, Public Eating (PSS‐HN, PE) [26]. The PE subscale has the following items: 0 = always eats alone to 4 = no restriction of place, food or companion (eats out at any opportunity).
- At T0, participants independently formulated one or more personal treatment goals. At T1 and T2, participants were asked to score their level of achievement using the following categories: “totally (95%–100%)”, “almost (65%–95%)”, “half (35%–65%)”, “partially (5%–35%)” or “not (0%–5%)”.

Clinician‐rated outcomes:

- Normalcy of diet was scored with food levels (adapted scale of the MDTP‐approach [23]), based on the most “safe to swallow” food during videofluoroscopy, as follows: 1 = moderately‐extremely thick liquids or liquidized or pureed foods (IDDSI level 3–4^1^ [27]), 5 mL; 2 = food level 1 with 10 mL; 3 = soft masticated (no dentition needed), for example banana; 4 = hard masticated (dentition needed), of which you can form a bolus, for example bread with crust; and 5 = hard masticated (dentition needed), of which you cannot form a bolus, for example raw carrot.
- Dysphagia severity was rated with the Australian Therapy Outcome Measures (AUS‐TOM [28]), ranging from 0 = profound swallowing/feeding impairment, to 5 = no swallowing impairment, scored for fluids and solids separately.
- Swallowing capacity was measured in two ways, if possible; (1) maximum swallowing speed (MSS) was recorded in mL/s [29, 30]; and (2) maximum swallowing volume (MSV) is the maximum amount of liquid (in milliliters) that could be swallowed in one single swallow [31].
- We assessed ability to manage solid food with the test of masticating and swallowing solids (TOMASS [32]) by recording duration in seconds, number of bites, masticatory cycles and swallows involved in eating of a 5 × 5 cm cracker.

### 2.5 Treatment

The ISR program is based on the established MDTP protocol [22, 23, 24], which focuses on intensive functional swallowing exercises [19], and was delivered by certified MDTP trainers. Only minor modifications were made to tailor the protocol to HNC‐related dysphagia, particularly the typical consequences of xerostomia. The program consisted of 15 weekday 60‐min sessions, supported by daily home practice. Therapy followed the MDTP food‐hierarchy‐structure, beginning with each participant's safest level and gradually progressing to higher food hierarchy levels, adjusting volume, consistency, intensity, and timing of swallowing. To accommodate xerostomia, patients were allowed to take small sips of water or consume a moisture containing item (e.g., tomato) between levels, and food levels were adjusted to better reflect xerostomia‐related difficulties such as bolus formation. Participants brought their own specific foods to practice with. After completing the program, they were instructed to continue daily practice at home using their most challenging food consistency.

Delivery of the ISR program was in‐person at the outpatient clinic, online or as a combination of both modalities, depending on the participants preference. Online participation required additional consent and caregiver presence during sessions, as the SLT was not physically present and therefore could not intervene in the event of, for example, a choking event. Caregivers received safety instructions during intake. Online sessions were conducted using secure platforms (Zaurus at Radboudumc, or HIX Chipsoft B.V. at NKI).

### 2.6 Statistical Analysis

Data were collected using Castor Electronic Data Capture (Ciwit B.V., version 1.6, study ID 48880AE0‐70B5‐4307‐A13A‐C2448B70D708) and analyzed using SPSS v27. Descriptive statistics were used for patient characteristics and for ordinal scales (PSS‐HN, food level and AUS‐TOM). For continuous data (VAS, MDADI, MSS, MSV and TOMASS), repeated‐measures ANOVA was performed, with Mauchly's test for sphericity. If sphericity was met, no correction was applied. Ordinal data were analyzes using the Friedman test, followed by Wilcoxon signed‐rank test for pairwise comparisons (T0–T1, T1–T2, and T0–T2). PSS‐HN, food level, AUS‐TOMs and goal achievement were presented as percentages per Likert category. Figures were created using R (v4.4.3, ggplot2).

## 3 Results

### 3.1 Participants

**FIGURE 1:**
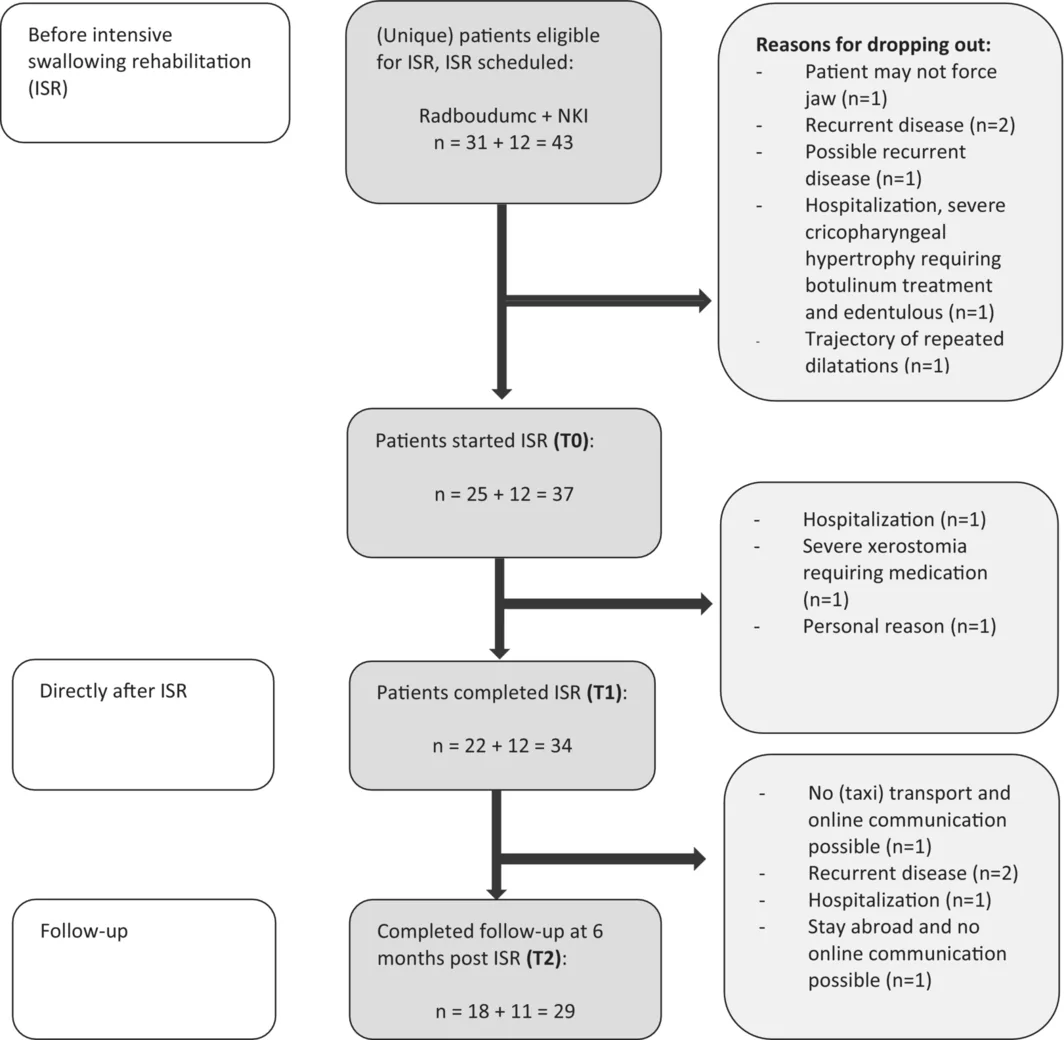
Flowchart of participants eligible for and completed (follow‐up of) intensive swallowing rehabilitation (ISR).

All participants who were offered ISR, were willing to participate. Forty‐three participants were initially included, of whom six dropped out before the start due to medical reasons (see Figure 1). During the program, three participants had to discontinue (two medical‐related, one personal reason).

Of the 34 participants who completed the ISR program, 24 participated in‐person, 3 online and 7 combined. Online participation was mainly chosen due to travel distance. At follow‐up, five participants were lost for various reasons (see Figure 1).

No severe adverse events, such as choking, occurred during this study in neither the in‐person nor the online sessions and no aspiration pneumonias were reported during follow‐up. Participant characteristics are presented in Table 1.

**TABLE 1:** Patient characteristics.

Characteristics	*n* = 34
Sex; women (*n*; %)	18 (53)
Age in years (mean; SD)	63 (9.8)
Months since medical treatment (median; IQR)	45 (12–116)
Tumor status	
Primary tumors	25 (74)
Secondary tumors	5 (15)
Recurrent tumors	4 (11)
Tumor location primary tumors (*n*; %)	
Oral cavity	3 (9)
Oropharynx	10 (29)
Nasopharynx	3 (9)
Hypopharynx	2 (6)
Larynx	5 (15)
Parotic gland	1 (3)
Unknown primary	1 (3)
T classification primary tumors (*n*; %)	
Tx	1 (3)
Tcis	1 (3)
T1	4 (12)
T2	5 (15)
T3	3 (9)
T4	9 (27)
Missing	2 (6)
*N* classification primary tumors (*n*; %)	
N0	6 (18)
N1	6 (18)
N2	8 (23)
N3	3 (9)
Missing	2 (6)
M classification primary tumors (*n*; %)	
M0	23 (68)
Missing	2 (6)
Medical treatment (*n*; %)	
Chemoradiation	14 (41)
Radiation	5 (15)
Surgery only	1 (3)
Surgery + adjuvant therapy	4 (11)

*Note:* Participants demographics: sex, age, months since medical treatment, tumor site, tumor‐node‐metastasis (TNM 7th and 8th edition, depending on year of diagnose) staging system and medical treatment details.

### 3.2 Patient‐Rated Swallowing Outcomes

**FIGURE 2:**
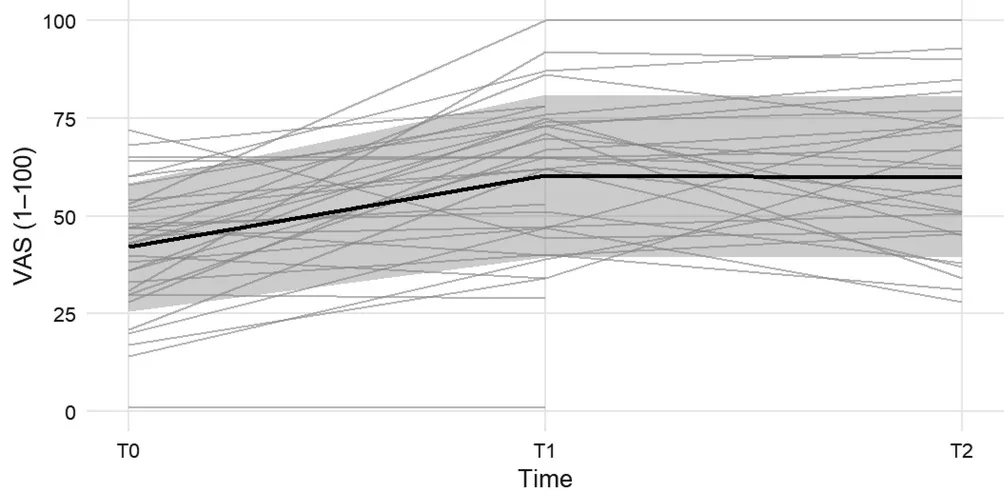
Visual analogue scale (VAS) scores (1–100) for individual participants at T0, T1 and T2. T0, T1, and T2 represent assessment time points: baseline (T0), directly post‐treatment (T1), and 6‐month follow‐up (T2). VAS = 1 = I can't swallow, 100 = I have no swallowing problems. The figure shows individual participant trajectories (thin lines), with the group mean represented by the thick black line and the standard deviation indicated by the light gray shaded area, and calculated in R. Total participants who completed intensive swallow rehabilitation is *n* = 34, *n* = 29 completed follow‐up at 6 months. At T0–T1, 1 VAS score is unintentionally missing and at T2 3 are unintentionally missing.

Table 2 gives an overview of patient and clinician‐rated outcomes and Figure 2 for the VAS scores for individual participants. After treatment (T1) participants rated significant less swallowing problems on the VAS (42 vs. 60; *p* < 0.001), a significantly higher dysphagia‐related quality of life (64 vs. 69; *p* = 0.037) and a significant better social participation rated on the PSS‐HN subscale PE (2.5 (IQR 2–3) to 3 (IQR 2–3), *p* < 0.001) (see Table 2). At follow‐up, the dysphagia‐related quality of life (MDADI) significantly increased from 69 (T1) to 73 at T2 (*p* = 0.037), while the other patient‐rated swallowing outcome measures sustained the 6 months follow‐up.

**TABLE 2:** Patient‐ and clinician‐rated swallowing outcomes.

*n* = 34		T0 mean SD	T1 mean SD	T2 mean SD	ANOVA (df) *F*	ANOVA (*η* ^2^ *ₚ*) *p*	Pairwise comparison (Bonferroni)^c^
Patient‐rated	VAS^a^ (1–100)	42.3 16.6	60.3 20.9	61.1 19.8	(2, 52) 17.5	(0.40) **< 0.001**	T0 versus T1; **< 0.001** T0 versus T2; **0.001** T1 versus T2; 1.000
	MDADI total (20–100)	64.0 13.0	69.1 11.9	72.7 11.8	(2, 56) 13.5	(0.33) **< 0.001**	T0 versus T1; **0.037** T0 versus T2; **< 0.001** T1 versus T2; **0.037**
		T0	T1	T2	Chi‐square	*p* value	Wilcoxon test
	PSS‐HN, PE	*n* (%)	*n* (%)	*n* (%)	11.662	**0.003**	T0 versus T1; **< 0.001** T0 versus T2; **0.010** T1 versus T2; 0.317
	0	4 (12)	1 (3)	1 (3)			
	1	3 (9)	4 (12)	2 (7)			
	2	10 (29)	6 (17)	4 (14)			
	3	15 (44)	19 (56)	15 (52)			
	4	2 (6)	4 (12)	7 (24)			
Clinician‐rated	Food‐level^b^	*n* (%)	*n* (%)	*n* (%)	37.932	**< 0.001**	T0 versus T1; **0.002** T0 versus T2; **< 0.001** T1 versus T2; 0.180
	1	12 (40)	1 (3)	0			
	2	14 (47)	2 (6)	3 (10)			
	3	4 (13)	20 (60)	14 (48)			
	4	0	2 (6)	6 (21)			
	5	0	8 (25)	6 (21)			
	AUS‐TOM solids	*n* (%)	*n* (%)	*n* (%)	21.233	**< 0.001**	T0 versus T1; **0.010** T0 versus T2; **< 0.001** T1 versus T2; **0.040**
	0	2 (5)	2 (5)	1 (3)			
	1	10 (30)	5 (15)	1 (3)			
	2	10 (30)	10 (30)	9 (31)			
	3	12 (35)	14 (41)	9 (31)			
	4	0	3 (9)	7 (25)			
	5	0	0	2 (7)			
	AUS‐TOM fluids	*n* (%)	*n* (%)	*n* (%)	7.156	**0.028**	T0 versus T1; 0.073 T0 versus T2; **0.028** T1 versus T2; 0.450
	0	0	0	0			
	1	10 (29)	7 (21)	3 (10)			
	2	6 (18)	6 (18)	4 (14)			
	3	8 (23)	8 (23)	9 (31)			
	4	7 (21)	9 (26)	9 (31)			
	5	3 (9)	4 (12)	4 (14)			

*Note:* Total participants who completed intensive swallow rehabilitation is *n* = 34, *n* = 29 completed follow‐up at 6 months. T0, T1, and T2 represent assessment time points: baseline (T0), directly post‐treatment (T1), and 6‐month follow‐up (T2). ANOVA indicates the results of repeated‐measures analysis of variance across these time points. Pairwise comparisons were adjusted using Bonferroni correction to control for multiple testing. Bold numbers are significant outcomes.

Abbreviations: 0, I always eat alone; 0, profound swallowing/feeding impairment; 1, I can't swallow; 100, I have no swallowing problems; 20, low dysphagia‐related QoL; AUS‐TOM, Australian therapy outcome measures; MDADI, MD Anderson dysphagia inventory; PSS‐HN, performance status scale for head and neck, public eating; VAS, visual analogue scale.

^a^ At T0–T1, 1 VAS score is unintentionally missing. At T2, *n* = 29 but 3 are unintentionally missing.

^b^ Food‐level was only scored in participants who could safely swallow foods as described in the Methods. At T0 food level was scored in 30/34 participants, at T1 33/34 and at T2 29/29.

^c^ Pairwise comparison is based on *n* = 26.

In total, 90 goals were set (median of 2 per participants (range 1–7)). Further analyses showed that most of the goals were normalcy of diet related, with a smaller subset addressing aspects of social participation. Examples of individual goals of participants were “To eat solid foods without the need of adding sauce or gravy.”; “Being able to drink without coughing.”; “To be able to join social activities which involve eating, without adjustments or fear of coughing.”

**FIGURE 3:**
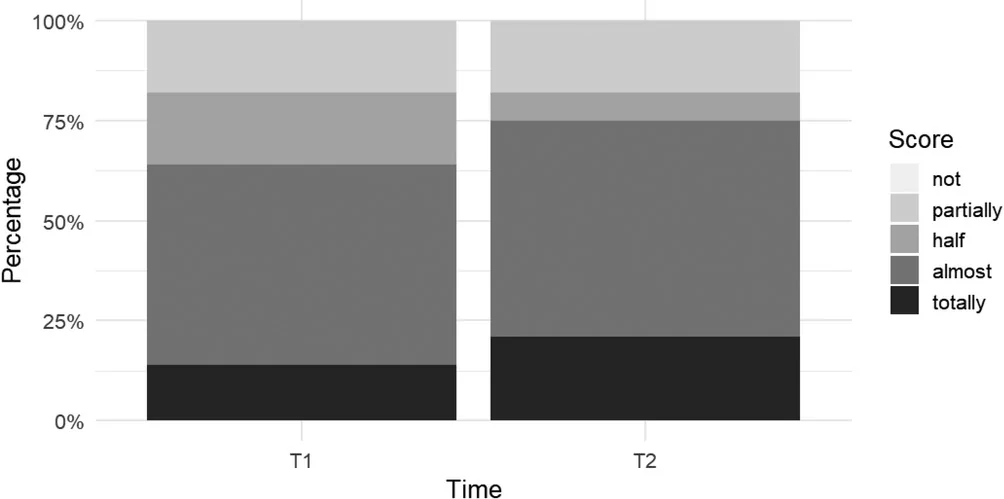
Percentage of goal achievement at T1 and T2. Total participants at (T1) is 34 and at T2 is 28 (1 unintentional missing score). T1 and T2 represent assessment time points: directly post‐treatment (T1), and 6‐month follow‐up (T2). At those timepoints, participants were asked to score their level of goal achievement using the following categories: “totally (95%–100%)”, “almost (65%–95%)”, “half (35%–65%)”, partially (5%–35%)” or “not (0%–5%)”. Bars represent the distribution of responses on the Likert scale, with percentages indicating the proportion of participants selecting each response category.

See Figure 3 for goal achievements at T1 and T2. At T1 14.7% of the goals was reached totally, at T2 this was 21.7%. Notably, the option “not achieved” was never selected at T1 or T2.

### 3.3 Clinician‐Rated Swallowing Outcomes

**FIGURE 4:**
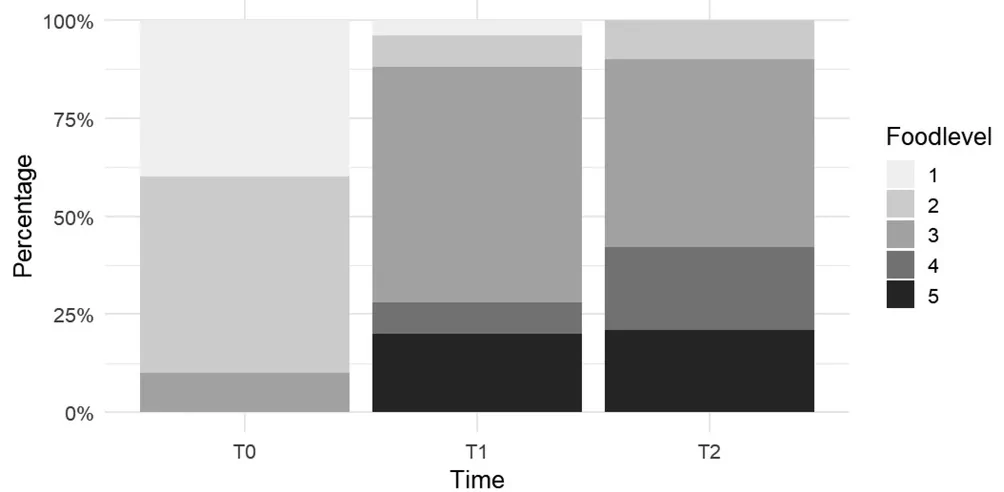
Percentages of food levels 1–5 at T0 (*n* = 30/34), T1 (*n* = 33/34), and T2 (*n* = 29/29). Food levels are scored as follows: 1 = moderately‐extremely thick liquids or liquidized or pureed foods (IDDSI 3–4), 5 mL, 2 = food level 1 with 10 mL, 3 = soft masticated (no dentition needed), 4 = hard masticated, dentition needed, of which you can form a bolus, 5 = hard masticated, dentition needed, of which you cannot form a bolus.

Food levels improved significantly after the ISR program (see Figure 4), with the median increasing from 2 (IQR 1–2.25) at T0, where 47% of participants scored level 2, to 3 (IQR 3–4.5) at T1, where 60% scored level 3. At T2, the median remained 3 (IQR 3–4), with 48% scoring level 3 (*p* = 0.002 for T0–T1; *p* = 0.180 for T1–T2; see Table 2). For AUS‐TOM solids, the median remained 3 across all time points, but the distribution shifted toward higher scores directly after ISR: at T0, 35% of participants scored level 3 or higher, compared to 50% at T1 and 63% at T2 (*p* = 0.010 for T0–T1; *p* < 0.001 for T0–T2; see Table 2).

For AUS‐TOM fluids, the median also remained 3, but the proportion of higher scores rose from 53% at T0 to 76% at T2 (*p* = 0.028 for T0–T2; see Table 2).

At the 6‐month follow‐up, MSS showed a significant difference in ANOVA (*p* = 0.016; see Table 3). However, after applying Bonferroni correction, this difference was no longer statistically significant. The volume of a single swallow (maximum swallowing volume; MSV) and TOMASS scores did not show significant change over time.

**TABLE 3:** Swallowing performance test outcomes.

	T0 mean SD	T1 mean SD	T2 mean SD	ANOVA (df) *F*	ANOVA (*η* ^2^ *ₚ*) *p*	Pairwise comparison (Bonferroni)
Maximum swallowing speed of liquids in mL/s (MSS)^a^	6.5 3.7	7.8 5.7	8.6 5.9	(2, 48) 4.55	(0.159) **0.016**	T0 versus T1; 0.230 T0 versus T2; 0.053 T1 versus T2; 0.509
Maximum swallowing volume in mL (MSV)^b^	7.4 9.6	9.7 11.6	6.4 8.2	(2, 36) 2.02	(0.101) 0.148	T0 versus T1; 0.479 T0 versus T2; 1.000 T1 versus T2; 0.215
TOMASS^c^ seconds	119.7 47.5	121.3 109.7	87.9 46.4	(2, 12) 1.35	(0.184) 0.296	T0 versus T1; 1.000 T0 versus T2; 0.068 T1 versus T2; 0.758
TOMASS number of bites	3.4 1.4	3.2 1.3	3.3 1.0	(2, 12) 0.37	(0.058) 0.697	T0 versus T1; 0.868 T0 versus T2; 1.000 T1 versus T2; 0.690
TOMASS masticatory cycles	102.1 50.9	87.7 35.1	81.7 27.7	(2, 12) 2.55	(0.298) 0.120	T0 versus T1; 0.730 T0 versus T2; 0.282 T1 versus T2; 0.878
TOMASS swallows	7.2 3.0	8.1 8.3	5.8 6.7	(2, 12) 0.721	(0.107) 0.506	T0 versus T1; 1.000 T0 versus T2; 0.925 T1 versus T2; 0.291

*Note:* Total participants who completed intensive swallow rehabilitation is *n* = 34, *n* = 29 completed follow‐up at 6 months. T0, T1, and T2 represent assessment time points: baseline (T0), directly post‐treatment (T1), and 6‐month follow‐up (T2). ANOVA indicates the results of repeated‐measures analysis of variance across these time points. Pairwise comparisons were adjusted using Bonferroni correction to control for multiple testing. Bold numbers are significant outcomes.

^a^ MSS was performed in 27 out of 34 participants at T0 and at T2 in 23 out of 29 participants who completed follow‐up. At T0, 7 missings were due to aspiration, 1 was unintentional.

^b^ MSV was performed in 27 out of 34 participants at T0 and at T2 in 19 out of 29 participants who completed follow‐up. 4 were unintentionally missing.

^c^ TOMASS: Due to technical difficulties, incorrect crackers were used during the process, resulting in several missings in the dataset. At T0, in 22 participants the correct cracker was used, and only 10 participants were able to perform the test. At T2, only 7 out of 18 were able to perform the test. Reasons for not being able to complete the test were xerostomia, aspiration, severe pharyngeal residue or the inability to chew.

## 4 Discussion

This study shows that daily ISR for three consecutive weeks is feasible, safe and effective for HNC survivors with chronic dysphagia. Participants showed significant improvements in normalcy of diet and dysphagia‐related quality of Life that sustained and further enhanced at 6 months follow‐up.

Despite the treatment intensity, all participants who were offered the ISR program, were willing to participate, and adherence during ISR was high: 92% completed all sessions (irrespective of treatment delivery, in‐person or online). This adherence is similar to the study of Charters et al. [19] on intensive swallowing rehabilitation in HNC patients, where all 10 participants completed the intervention. Drop‐out in our study during ISR was unrelated to the (intensity of the) intervention (medical (*n* = 2) or personal reasons (*n* = 1)). Contributing factors of the high adherence might be the chronicity and severity of swallowing complaints. Constantinescu et al. [33] found that patients more than 2 years post‐treatment showed higher adherence to home‐based swallowing therapy than those treated more recently. This may reflect greater motivation after living longer with dysphagia or fewer medical appointments and complications interfering with participation. In addition, SLT in the current study considered patients to be fit for the ISR program, which could have added to the adherence, and many participants reported noticeable improvement during week one, likely boosting motivation and confidence.

Feasibility was further supported by the absence of any adverse events (e.g., severe choking) or aspiration pneumonias during follow‐up, indicating that ISR is safe, well‐tolerated, and suitable for broader clinical implementation.

Directly after ISR, all patient‐rated scores improved. Although the outcomes are not directly comparable with previous studies on intensive swallowing treatment for chronic dysphagia [18, 19], similar positive trends in patient‐rated outcomes were reported. Nearly two‐thirds of our participants scored their goals as totally or almost achieved, which is notable given the median of 4 years post medical treatment, a period sometimes characterized by increased radiation‐induced fibrosis and development of self‐taught compensation or avoidance strategies. Meaningful improvement suggests it is never too late to initiate such an intervention. Daily practice may play a key role in establishing more functional habits, particularly when longstanding compensations are present. Govender et al. [34] argue that the behavioral strategy of habit formation, developed through repeated behavior in a consistent context that becomes less reliant on conscious intention, may drive long‐term improvements. Since ISR is based on functional eating during daily meals, the exercises may naturally become routine, supporting sustained, or even progressive, improvements. Behavioral change may be less likely with weekly or low‐frequency sessions, as longer intervals give patients more opportunity to fall back to familiar strategies rather than strengthen more efficient swallowing patterns. These findings also raise the question of whether earlier intervention could lead to greater or faster improvements. However, optimal timing and frequency for intervention remains to be determined.

Although functional outcomes like food level improved directly post‐treatment, impacting daily food choices and normalcy of diet, objective surrogate swallowing performance tests (MSS and TOMASS) did not show measurable changes, suggesting that improvements were driven more by behavioral adaptation and self‐confidence than by physiological changes. As Baijens et al. [35] state, counseling and patient education, both key components of the ISR program, play an important role in increasing confidence. In addition, these surrogate measures seem hardly sensitive to functional swallowing improvements in our HNC patients. For example, patients may replace the need to spit out or evacuate the bolus with additional swallows, improving social eating without altering total eating time. Their validity as outcome indicators is further limited by the fact that several participants could not perform the tests due to aspiration, residue, or xerostomia. Similar to Pedersen et al. [36], we found patient‐reported improvements were strongly aligned with diet restrictions but poorly correlated with clinical assessments. Patients gradually learn to swallow safely and effectively, and the initial boost of confidence combined with consistent use of newly learned swallowing techniques during daily eating may facilitate long‐term improvements. A process that current surrogate swallowing tests fail to capture, reflecting the broader challenge noted by Kraaijenga et al. [37], regarding the “lack of consensus” in dysphagia assessment.

Although the long‐term effects of swallowing therapy in chronic HNC‐related dysphagia remain largely unknown, this study suggest promising potential for ISR. At 6‐month follow‐up, improvements were sustained, with better patient‐rated (MDADI) and clinician‐rated outcomes (AUS‐TOM for solids). The 8.7‐point increase in MDADI from T0 to T2 falls just below the 10‐point threshold for clinical relevance [38], yet still suggests some meaningful improvement in dysphagia‐related quality of life. Objective surrogate swallowing tests showed no significant long‐term change after Bonferroni correction; the small MSS improvement (2.1 mL/s) remained well below normal values and the 5 mL/s threshold for clinical relevance [39]. Beyond improvements in diet and dysphagia‐related quality of life, the increase in AUS‐TOM scores for fluids at 6 months suggests possible gains in swallowing safety. However, without instrumental assessment, (silent) aspiration cannot be ruled out.

This study has several limitations. First, the absence of a control group limits attribution of improvements solely to ISR, meaning that other influences or placebo effect cannot be ruled out. Second, because spontaneous recovery is unlikely in people with chronic dysphagia, we assumed that patients could serve as their own control, without measuring the stability of their functioning pre‐treatment. Although all our participants had dysphagia longer than a year post medical treatment (median 45 months), results would have been more robust if we would have been able to demonstrate stable baselines or interpreting pre‐treatment recovery of deterioration (due to for example late radiating‐associate toxicity). Third, although patient characteristics (e.g., tumor location, tumor stage, medical treatments) may influence outcomes, subgroup analyses were not feasible due to limited sample sizes within these categories. Thus, despite the relatively large overall HNC sample, its heterogeneity restricts the ability to draw meaningful conclusions for specific subgroups. Fourth, the sample included 53% women, contrasting with the typical male‐dominated HNC population [40, 41], potentially reflecting selection bias related to differences in symptom awareness or opportunity to engage in such an intensive program. This discrepancy may limit the generalizability of our findings to the broader HNC population. Fifth, goals were freely formulated without categorization (such as normalcy of diet, swallowing safety, participation). While classification of goals was not a study objective, future studies should classify goals to identify which are most achievable. Sixth, objective surrogate swallowing tests appeared insufficiently sensitive to functional improvements; incorporating videofluoroscopy in future research may better distinguish functional from physiological changes. Seventh, adverse effects were not systematically monitored, as no events were anticipated based on prior clinical experience. Nevertheless, applying a structured system such as Common Terminology for Adverse Events [CTCAE] [42] could have provided additional insight into mild symptoms and represents valuable consideration for future studies. Finally, home practice during follow‐up was not systematically monitored. Although this was not the purpose of this study, tracking continued use of learned techniques could provide valuable insights.

Typically, the strength of the study was that it was well‐structured and finished with minimal dropouts, strengthening the validity of the reported results.

The results of this study generate several research questions for future investigation. Larger studies incorporation patient characteristics could improve prediction of eligibility and outcomes, while the optimal timing of ISR remains unclear. Although some eligible participants dropped out for urgent medical reasons, earlier intervention (< 1 year post medical treatment) may prevent ineffective strategies, anxiety, or unnecessarily restrictive food consistencies. This also highlights the need for structured (medical) follow‐up and screening tools to identify ISR candidates, as well as how such follow‐up could align with or complement existing preventive swallowing rehabilitation strategies [14].

Broader implementation faces challenges including program intensity, SLT availability, patient and caregiver burden, and reimbursement. Future health economic evaluations, including cost‐effectiveness and cost‐utility studies, will be essential to support long‐term program sustainability and to assess for example whether a lower‐intensity schedule might offer a more feasible yet effective option.

## Conflicts of Interest

The authors declare no conflicts of interest.

## Data Availability

The data that support the findings of this study are available on request from the corresponding author. The data are not publicly available due to privacy or ethical restrictions.

## Endnotes

^1^
International dysphagia diet standardization initiative (IDDSI), global standardized terms and definitions for texture‐modified foods and liquids, whereas thin liquids correspond with IDDSI 0, thick liquids with IDDSI 3 and all solid foods with IDDSI 7.
